# A Cost-Variation Analysis of Drugs Available in the Indian Market for the Management of Diabetic Nephropathy

**DOI:** 10.7759/cureus.29942

**Published:** 2022-10-05

**Authors:** Manjari Advani, Rajmohan Seetharaman, Manish Pawar, Bakul Naik

**Affiliations:** 1 Pharmacology, Lokmanya Tilak Municipal Medical College and General Hospital, Mumbai, IND

**Keywords:** developing country, diabetic kidney disease, price variation, drug availability, pharmacoeconomic analysis

## Abstract

Background

Diabetic nephropathy is associated with polypharmacy and increased out-of-pocket expenditure for the patients. Multiple brands of each prescribed drug are available in the market. Hence, there is a need to evaluate the cost variation of the available brands of prescribed drugs.

Methodology

All drugs prescribed to the 282 patients with diabetic nephropathy from our previous cross-sectional observational drug utilization study were included. Data regarding the cost of various brands of the prescribed drugs were obtained from Current Index of Medical Specialities (CIMS) android application version 3.1.2 and Indian online pharmacies. The percentage price variation and cost ratio for these drugs were determined. A correlation analysis was conducted between the number of brands and percentage price variation.

Results

A high percentage price variation (>1,000%) and cost ratio (>10) was observed for 19 out of 39 drugs that were evaluated. The highest price variations were seen with amlodipine (16,799%), metformin (11,240%), and glimepiride (10,525%). The highest cost ratios were seen with amlodipine (168), metformin (113.40%), and glimepiride (106.25%). There was a negligible correlation between the number of brands and percentage price variation.

Conclusions

The above findings indicate that drug price variations need to be monitored more strictly. The present study may aid physicians in understanding the degree of price variation among medications used for the treatment of diabetic nephropathy, thereby necessitating the selection of a P-drug to increase the affordability of drugs for patients.

## Introduction

According to the International Diabetes Federation (IDF) 2022 statistics, 8.3% of Indian people have diabetes mellitus (DM) [1]. About 40% of diabetes patients are at risk for developing diabetic nephropathy, which is the leading global contributor to end-stage renal disease (ESRD). According to a 2019 study, diabetes-related chronic kidney disease (CKD) is now the world’s seventh most common non-communicable disease, the sixth leading cause of disability, and the fourth leading cause of mortality [2].

Recent healthcare-related claims data reported that diabetic nephropathy is associated with higher healthcare expenditure and health resource utilization. The out-of-pocket (OOP) expenses of patients rise due to these costs. The market’s wide price range for the brands of prescription medications could further boost the cost to the patient [3]. The National Pharmaceutical Pricing Authority (NPPA) implemented the Drug Price Control Order (DPCO) in India to control the cost of medicines sold in the country. It must be determined whether there are significant pricing variations in the Indian market following the introduction of the DPCO [4].

Patients with diabetic nephropathy are prescribed medications for indications, including antidiabetics, antihypertensives, antimicrobials, statins, nutritional supplements, and many others [5]. This leads to polypharmacy and increased expenditure on medications for patients. Previous cost-variation studies have analyzed the above classes of drugs for their individual indications. However, none of these studies have considered a comprehensive evaluation of all medications given to patients with diabetic nephropathy [6,7].

Therefore, we undertook this pharmacoeconomic study to evaluate the cost variation and cost ratio for all medications available in the Indian market prescribed to patients with diabetic nephropathy in our previous drug utilization study [5].

## Materials and methods

Study design

An analytical cross-sectional study was conducted. Ethical approval (IEC/375/19) for the study was obtained from the Institutional Ethics Committee of Lokmanya Tilak Municipal Medical College and General Hospital, Mumbai. We previously conducted a cross-sectional observational drug utilization study in 282 patients with diabetic nephropathy. This study evaluated the World Health Organization’s (WHO) core prescribing, dispensing, and patient care indicators in these patients. The drugs prescribed in this study (antidiabetics, antihypertensives, antimicrobial, and other relevant drugs) were included for cost analysis in this study [5]. The cost of various oral or parenteral, or both drug formulations, was collected. The cost of each unit of daily defined dose (DDD) was compared. Units were defined based on the methodology employed in previous cost-variation analysis studies wherein 1 unit was equivalent to 10 doses of DDD (1 unit = 10 tablets/10 doses of injectables) [8].

Study materials

The Current Index of Medical Specialities [CIMS] android application (July 2022) containing the latest updates (version 3.1.2) and Indian online pharmacies were used to extract data regarding the prices of various brands of the drugs mentioned above. Data were collected for all available strengths and dosage forms. The NPPA website was used to gather information about the generic drug pricing set by the NPPA under the DPCO [9]. The Consolidated Health Economic Evaluation Reporting Standards (CHEERS) 2022 guidelines were used for the reporting of the study.

Definitions

DDD, as standardized by the WHO Collaborating Centre for Drug Statistics and Methodology, is the maintenance dose of a drug used for a particular indication. Cost in terms of per unit of DDD and the maximum and minimum cost per unit of DDD were evaluated. The cost ratio is the ratio of the costliest to the cheapest brand [8]. Percentage price variation (PPV) was calculated using the following formula: PPV [8] = [(Price of the most expensive brand - Price of the least expensive brand) × 100]/Price of the least expensive brand.

Statistical analysis

A correlation analysis between the number of brands and percentage price variation was performed. The data were evaluated for normality using the Shapiro-Wilk test, and Pearson’s or Spearman’s correlation was applied accordingly. A correlation coefficient (r) of 0-1 was considered a positive correlation. The confidence intervals were also obtained along with correlation analysis.

## Results

Prescribed classes of drugs

A total of 39 drugs were included in the study consisting of 13 (33.33%) antidiabetics, 14 (35.90%) antihypertensives, four (10.26%) antimicrobials, and eight (25.51%) other drugs consisting of antiplatelets, fibrinolytics, nitrates, statins, antimicrobials, haematopoetic agents, and metabolic modulators (Table 1).

**Table 1:** Prescribed classes of drugs with WHO ATC/DDD value. *DDD value of the drug is not available. Hence, the doses prescribed in our previous study are considered for evaluation [5]. ATC: anatomical therapeutic chemical; DDD: defined daily dose; PPAR γ: peroxisome proliferator-activated receptor gamma; GLP-1: glucagon-like peptide 1; DPP-4: dipeptidyl peptidase 4; SGLT-2: sodium/glucose cotransporter 2; ACEI: angiotensin-converting-enzyme inhibitors; ARB: angiotensin II receptor blockers; CCB: calcium channel blockers

Class	Drug	ATC No	1 DDD value
Antidiabetics			
Insulins	Plain insulin	A10AB04	40 IU
	Mixtard insulin H30/I70	A10AD01	40 IU
Sulfonylureas	Gliclazide	A10BB09	60 mg
	Glimepiride	A10BB12	2 mg
Biguanides	Metformin	A10BA01	2,000 mg
PPAR γ	Pioglitazone	A10BG03	30 mg
α-glucosidase inhibitor	Voglibose	A10BF03	0.6 mg
GLP-1 analog	Liraglutide	A10BJ02	1.5 mg
DPP-4 inhibitors	Sitagliptin	A10BH01	100 mg
	Linagliptin	A10BH05	5 mg
	Teneligliptin*	A10BH08	N/A – 20 mg
SGLT-2 inhibitors	Dapagliflozin	A10BK01	10 mg
	Empagliflozin	A10BK03	17.5 mg
Antihypertensives			
ACEI	Enalapril	C09AA02	10 mg
	Ramipril	C09AA05	2.5 mg
ARB	Losartan	C09CA01	50 mg
	Telmisartan	C09CA07	40 mg
CCB	Amlodipine	C08CA01	5 mg
	Nifedipine	C08CA05	30 mg
	Cilnidipine	C08CA14	10 mg
	Efonidipine*	C08	N/A – 10 mg
Loop diuretics	Furosemide	C03CA01	40 mg
β1 blocker	Metoprolol tartrate	C07AB02	150 mg
	Bisoprolol	C07AB07	10 mg
α + β blocker	Labetalol	C07AG01	600 mg
α_1_ blocker	Prazosin	C02CA01	5 mg
α_2_ agonist	Clonidine	C02AC01	450 mcg
Antimicrobials			
Cephalosporins	Ceftazidime	J01DD02	4 g
	Ceftriaxone sodium	J01DD04	2 g
	Cefixime	J01DD08	400 mg
Fluoroquinolones	Ciprofloxacin	J01MA02	0.8 g
Other drugs			
Antiplatelet	Aspirin	N02BA01	3 g
Fibrinolytic	Clopidogrel	B01AC04	75 mg
Statins	Atorvastatin	C10AA05	20 mg
	Rosuvastatin	C10AA07	10 mg
Nitrates	Isosorbide mononitrate	C01DA14	40 mg
Hematopoietic agents	Erythropoietin	B03XA01	1 IU
Calcium supplements	Calcium acetate	V03AE07	6 g
Electrolytes	Sodium bicarbonate*	B05XA02	N/A – 300 mg

Number of brands

Among the antidiabetics, glimepiride had the highest number of brands (431), followed by teneligliptin (351), and pioglitazone (288). The lowest number of brands were seen with liraglutide (2), followed by empagliflozin (8). Among the antihypertensives, losartan had the highest number of brands (280), followed by amlodipine (252), and cilnidipine (193). The lowest number of brands were seen with efonidipine (2) and clonidine (6). Among the antimicrobials, cefixime (205) had the highest number of brands, and ceftazidime (141) had the lowest number of brands. Among the other drugs, atorvastatin had the highest number of brands (634), followed by rosuvastatin (110), and clopidogrel (99). Calcium acetate (2) and isosorbide mononitrate (38) had the least number of brands (Table 2).

**Table 2:** Cost-variation analysis among prescribed drugs per unit of defined daily dose for diabetic nephropathy patients. At the time of the calculation, 1 USD was 80 INR. *Includes all brands and branded generics available in the Indian market. **Calculation is done as per WHO ATC/DDD; however, much higher doses are prescribed in the Indian setup (dose: 4,000 I/U: Maximum cost: 1,677 INR, Minimum cost: 990 INR). ^#^Maximum values. ^Minimum values. DPCO: Drug Price Control Order

Serial number	Drug	Price fixed by DPCO (INR)	Number of brands*	Minimum price per DDD* (INR)	Maximum price per DDD (INR)*	Cost ratio of brands*	Percentage price variation* (%)
Antidiabetics							
	Plain insulin	167.1	10	157	174	1.11^	11^
	Lupinsulin H30/I70	167.1	10	157.84	569	3.61	261
	Gliclazide	N/A	210	60.50	276	4.56	356
	Glimepiride	63.3	431^#^	8	850	106.25^#^	10,525^#^
	Metformin	84.8	257	20	2,268	113.40^#^	11,240^#^
	Pioglitazone	N/A	288^#^	10	890	89.00^#^	8,800^#^
	Voglibose	N/A	123	30	1,131.75	37.73	3,673
	Liraglutide	N/A	2^	1,185	1,331	1.12^	12^
	Sitagliptin	N/A	10	95	820	2.63	163
	Linagliptin	N/A	16	250	658	8.63	763
	Teneligliptin	N/A	351^#^	22	343	15.59	1459
	Dapagliflozin	N/A	137	88.5	812	9.18	818
	Empagliflozin	N/A	8^	126	574	4.56	356
Antihypertensives							
	Enalapril	71.2	163	40	310	7.75	675
	Ramipril	43.45	135	7	211	30.19	2,919
	Losartan	N/A	280^#^	12	520	43.33^#^	4,233^#^
	Telmisartan	N/A	217^#^	15	530	35.33	3,433
	Amlodipine	28.9	252^#^	3.50	588	168.00^#^	16,700^#^
	Nifedipine	28.5	58	30.60	127.53	4.17	317
	Cilnidipine	41.1	193	40	382	9.55	855
	Efonidipine	N/A	2^	30	32.96	1.10^	10^
	Furosemide	8.3	19	5.40	111	20.56	1,956
	Metoprolol	N/A	90	18	1,113.90	61.88^#^	6,088^#^
	Bisoprolol	N/A	31	80	297.18	3.71	271
	Labetalol	N/A	22	798.60	7500	9.39	839
	Prazosin	N/A	24	106	544	5.13	413
	Clonidine	N/A	6^	103.50	297	2.87^	187^
Antimicrobials							
	Ceftazidime	213	141^	100	2996	29.96^	2,896^
	Ceftriaxone sodium	324.6	144	17.80	778	43.71	4,271
	Cefixime	245	209^#^	30.04	1200	39.95	3,895
	Ciprofloxacin	64.8	163	0.01	0.94	71.52^#^	7,052^#^
Others							
	Aspirin	N/A	86	0.19	8.92	46.75^#^	4,575^#^
	Clopidogrel	77.2	99^#^	5	446	89.20^#^	8,820^#^
	Atorvastatin	147.5	634^#^	0.45	44.74	99.42^#^	9,842^#^
	Rosuvastatin	N/A	110^#^	2.50	62.40	24.96	2396
	Isosorbide mononitrate	N/A	38^	47.72	244.60	5.13	413
	Erythropoietin**	N/A	51	0.25	0.42	1.69^	69^
	Calcium acetate	N/A	2^	0.43	0.52	1.21^	21^
	Sodium bicarbonate	N/A	59	18.60	45.00	2.42	142

Cost ratio

Among antidiabetics, the highest cost ratio was seen with metformin (113.40), followed by glimepiride (106.25), and pioglitazone (89). The lowest cost ratio was seen with plain insulin (1.11) and liraglutide (1.12). Among antihypertensives, the highest cost ratio was seen with amlodipine (168), followed by metoprolol (61.88) and losartan (43.33). The lowest cost ratio was seen with efonidipine (1.10) and clonidine (2.87). Among antimicrobials, the highest cost ratio was seen with ciprofloxacin (71.5), and the lowest cost ratio was seen with ceftazidime (29.96). Among the other drugs, the highest cost ratio was seen with atorvastatin (99.42), followed by clopidogrel (89.20), and aspirin (46.75). The lowest cost ratio was seen with calcium acetate (1.21) and erythropoietin (1.69) (Table 2).

Percentage price variation

Among antidiabetics, the highest percentage price variation was seen with metformin (11,240%), followed by glimepiride (10,525%), and pioglitazone (8,800%). The lowest percentage price variation was seen with plain insulin (11%) and liraglutide (12%). Among antihypertensives, the highest percentage price variation was seen with amlodipine (16,700%), followed by metoprolol (6,088%), and losartan (4,233%). The lowest percentage price variation was seen with efonidipine (10%) and clonidine (187%). Among antimicrobials, the highest cost ratio was seen with ciprofloxacin (7,052%), and the lowest cost ratio was seen with ceftazidime (2,896%). Among the other drugs, the highest cost ratio was seen with atorvastatin (9,842%), followed by clopidogrel (8,820%), and aspirin (4,575%). The lowest cost ratio was seen with calcium acetate (21%) and erythropoietin (69%) (Table 2).

Correlation analysis

**Figure 1:**
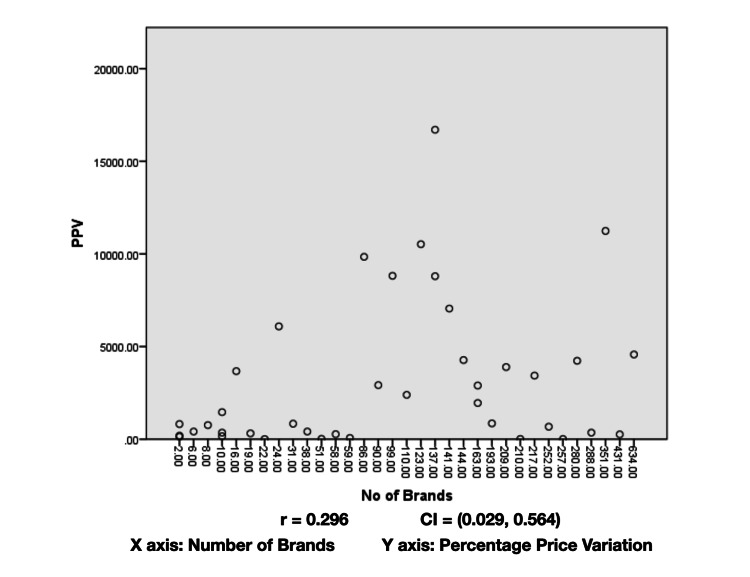
Correlation analysis between the number of brands and the percentage price variation of the prescribed drugs.

The number of brands did not show a normal distribution of the Shapiro-Wilk test (p < 0.001). Spearman’s correlation analysis between the number of brands and the percentage price variation showed a negligible correlation (0.296) (Figure 1).

## Discussion

This study is the first cost-variation analysis conducted in India that thoroughly assesses the most common medications prescribed to patients with diabetic nephropathy in a government-run hospital. It provides an idea regarding the overall cost variations in drug prices for these patients (Table 1). A cost-variation analysis of the most commonly prescribed drugs gives a direct estimate of the cost burden to the patient. Hence, the study does not include antidiabetics, antihypertensives, or antimicrobials that were not prescribed to patients in our previous study [5].

Despite the implementation of DPCO in 2013, it was observed that it was not being adhered to. The maximum cost for the drugs exceeded the DPCO-set price limit (Table 2)\. This suggests that the DPCO has to be implemented with greater rigor. A more refined method needs to be carried out to obtain a ceiling price for each drug so that the order can be executed with feasibility. The government had started an initiative to obtain the price data of the drugs sold in India from all existing manufacturers. This process needs to be sped up, as mentioned by Ray et al. [8]. It was also observed that a DPCO was not available for some of the commonly prescribed drugs, such as teneligliptin, losartan, telmisartan, and many more. This indicates that the National List of Essential Medicines (NLEM) must be updated more regularly [8].

Many drugs showed a high percentage price variation and cost ratio, which is consistent with the findings of other similar Indian studies carried out for different indications (Table 2) [6-8]. Physicians need to be aware of drugs with a relatively higher cost variation, such as metformin, glimepiride, amlodipine, metoprolol, atorvastatin, clopidogrel, and many more. Some studies have recommended that a doctor’s manual on drug pricing should be made available to practicing physicians to facilitate the above process [8]. Increased knowledge of the above may help physicians select their P-drug based on the Safety, Tolerability, Efficacy, and Price (STEP) criteria [10]. The government needs to promote the use of generic drugs to reduce the overall cost to patients. However, the poor perception of generic drugs among patients and physicians provides a major hurdle to the latter [8].

A negligible correlation was observed between the number of brands of a drug and its percentage price variation (Figure 1). Previous studies have reported a weak correlation between these two parameters for the other indications. The present results affirm the previous findings that an increased number of brands does not impact the price variation of drugs [8].

## Conclusions

The findings of the study indicate that the DPCO needs stricter implementation. The cost variation among drugs needs to be reduced by the measures provided above to reduce the OOP expenditure to patients. This study may assist physicians treating patients with diabetic nephropathy, with the selection of their P-drug from several treatment classes in a market with a high degree of drug price variation. This can additionally boost patient access to affordable drugs.
